# Taekwondo Training Improves Mood and Sociability in Children from Multicultural Families in South Korea: A Randomized Controlled Pilot Study

**DOI:** 10.3390/ijerph15040757

**Published:** 2018-04-16

**Authors:** Hee-Tae Roh, Su-Youn Cho, Wi-Young So

**Affiliations:** 1 Department of Physical Education, College of Arts and Physical Education, Dong-A University, Busan 49315, Korea; ilove9826@hanmail.net; 2 Department of Taekwondo, Youngsan University, Yangsan-si 50510, Korea; csy@swu.ac.kr; 3 Sports and Health Care Major, College of Humanities and Arts, Korea National University of Transportation, Chungju-si 27469, Korea

**Keywords:** taekwondo, sociality, mood state, multicultural family, children

## Abstract

Purpose: Children from multicultural families face physical, social, mental, and intellectual hurdles; however, relative interventions are lacking in South Korea (hereafter Korea) in this regard. The purpose of this study was to investigate the effects of regular Taekwondo (TKD) training on physical fitness, mood, sociability, and cognitive functions in these children. Methods: This study included 30 children from multicultural families in Korea who were randomly assigned to a TKD group (*n* = 15) and control group (*n* = 15). The children in TKD group underwent 16 weeks of TKD training once a week for 60 min. Each participant underwent a basic fitness test and sociability questionnaire before and after the intervention. Furthermore, we examined the changes in the mood and cognitive function by determining the profile of mood states (POMS), and Stroop color and word test, respectively. Results: Results of the Stork test of balance were significantly higher in the TKD group after intervention (*p* < 0.05). In terms of sub-variables, POMS, tension, and depression scores were significantly lower (*p* < 0.05) after the intervention, while the vigor score was significantly higher in the intervention group than those in the control group (*p* < 0.05). Furthermore, sociability and ‘being left out’ score, a sub-variable of sociability, was significantly lower (*p* < 0.05) after the intervention, while sociability score was significantly higher (*p* < 0.05). Conclusions: Our findings suggest that participation in regular TKD training can be effective for balanced improvements in variables of basic fitness and that it exerts a positive effect on the mood and development of sociability.

## 1. Introduction

The number of children from multicultural families due to international marriages and immigrant workers is rapidly increasing in South Korea (hereafter referred to as Korea). In 2016, the number of children from multicultural families was estimated to be approximately 200,000, a significant increase from the estimated 25,000 in 2006 [1,2]. Furthermore, it was estimated that 113,506 were pre-school children (age < 6 years, 56.4%), 56,768 were elementary school students (aged 7–12 years, 28.3%), and 31,059 were middle- and high school students (aged 13–18 years, 15.4%) [2], indicating that the number of children from multicultural families in Korea will increase exponentially over the next 5–10 years. However, it has been reported that multicultural families and the increasing number of children are likely to experience severe difficulties in comparison with mono-cultural Korean children due to different cultural and historical backgrounds, language, culture, and educational methods, communication problems, and low socioeconomic status of the parents [1].

According to recent studies, bi-ethnic adolescents (adolescent children born of marriages of migrants or naturalized Koreans) can be vulnerable to violence at school as Koreans have strong intolerance towards other ethnicities, suggesting that experiencing violence at school and being left out will be closely related to depression [3,4]. Furthermore, Kim et al. suggested that the low acculturation level of immigrant children and adolescents is connected to behavioral problems, such as adolescent delinquency [5]. Moon and An further reported that these children are likely to demonstrate hostility, phobic anxiety, and angering, which are tendencies of children from multicultural families to express anger [6]. The problems faced by children from multicultural families in Korea are not limited to social and emotional aspects. For example, it has been reported that children from multicultural families are vulnerable to verbal development delay because of communication problems with mothers who have difficulty communicating in Korean, resulting in limitations in communication with their peers at school, thus exerting a bad influence on cognitive development and comprehension [7].

Among the many combat sports, the Korean martial art of Taekwondo (TKD) is a popular sport that is characterized by high speed, high tension, and full-contact combat. It has been estimated that more than eighty million people worldwide participate in this sport [8,9,10] with a wide range in the age of the participants in more than 180 countries, especially children and adolescents. It has also been reported that the number of children participating in TKD is increasing globally [11,12,13]. Several recent studies have reported the significant health benefits of regular TKD training [11,13,14,15]. For example, Fong and Ng (2011) critically reviewed 23 TKD-related papers and investigated the effect of regular TKD training on the improvements in physical fitness [11]. Their analyses revealed no conclusive results in relation to the improvements in anaerobic fitness or muscle strength; however, the results did indicate improvements in aerobic capacity, body composition, fat loss, and flexibility [11]. Since then, Kim et al. reported that just 12 weeks of TKD training can induce positive improvements in terms of body composition and flexibility in female adolescents as well as a gain in skeletal muscle fitness [14]. Furthermore, Lee and Kim reported that TKD training can be effective in improving physical fitness and growth and development in children [13]. By using functional magnetic resonance imaging to observe the brains of children who participated in regular TKD training, Kim et al. reported a significant improvement in the activation of the brain, implying that TKD training can have a positive effect on the growth and development, fitness, and certain aspects of the health of the brain including cognitive function [15].

However, most TKD training-related studies have been limited to the physiological changes in children, although such studies have confirmed a positive correlation between exercise/physical activity and cognition, academic achievements, behavior, and psychosocial function in children [16]. Thus, the present study aimed to investigate the effect of TKD training on physical fitness, mood, sociability, and cognitive function in children from multicultural families who have relatively poor health-related factors compared with mono-cultural Korean children.

## 2. Methods

### 2.1. Subjects

This study included 30 children from multicultural families in Korea. Their fathers were Korean and their mothers were Chinese, Vietnamese, or Japanese. The children were randomly assigned to a control group (*n* = 15) and TKD group (*n* = 15). They attended elementary school at grades 4–6 and did not participate in regular exercises except for the physical education class at school (2–3 times a week) and did not have any mental or physical illnesses. All the participants were born and raised in Korea. The characteristics of the subjects are shown in Table 1. We excluded children who already had experience with TKD or lacked the ability to communicate in interviews. The study purpose, method, and procedures were explained to the parents of all subjects and they signed a consent form that included information such as voluntary withdrawal of participation. The study protocol was approved by the Institutional Review Board of Dong-A University (ID: 2-104709-AB-N-01-201609-HR-035-06).

### 2.2. Pre-Interventional and Post-Interventional Testing

Testing was conducted before (pre-) and after (post-) 16 weeks of intervention by selecting indices that could analyze the physique, fitness, mood state, sociability, and cognitive function of each subject. 

Physique was assessed by the height, weight, body mass index (BMI), and percentage of body fat. Height was measured using a stadiometer (SECA213, SECA, Hamburg, Germany), while weight, BMI, and the percentage of body fat were measured using a body composition analyzer (Inbody720, Biospace, Seoul, Korea).

Physical fitness was measured by cardiorespiratory endurance (VO_2_max), strength (grip strength and back strength), flexibility (sit-and-reach), power (Sargent jump), and balance (Stork test). Specifically, the cardiorespiratory endurance was measured by estimating VO_2_max with the Nemeth protocol on a treadmill (Q65, Quinton, Milwaukee, WI, USA) while wearing a wireless heart rate measuring equipment (Polar-a5, Polar, Kempele, Finland) [17]. The formula used for cardiac endurance was as follows: [VO_2_max = −1772.81 + 318.64 × gender (girl = 0, boy = 1) + 18.34 × weight (kg) + 24.45 × height (cm) − 8.74 × 4 min heart rate − 0.15 × weight (kg) × heart rate difference + 4.41 × treadmill speed (mph) × heart rate difference]. Grip and back strength were measured twice up to tenth of a kg using a digital grip (GRIP-D, Takei, Tokyo, Japan) and back strength (BACK-D, Takei, Tokyo, Japan) measuring equipment, respectively, and the highest score was recorded for analysis. Sit-and-reach, Sargent jump, and Stork test were measured twice using basic fitness measuring equipment (Helmas-III, O2run, Seoul, Korea), and the best score was recorded for analysis.

The mood state was estimated using the Korean Version of the Profile of Mood State-Brief (K-POMS-B) described by Yeun and Shin-Park, which is an adaptation of the profile of mood states (POMS) originally described by McNair et al. for Korea, and verified its reliability and validity [18,19]. This questionnaire consists of a total of 30 items, which are rated on a 5-point Likert scale (0 = not at all, 1 = a little, 2 = moderately, 3 = quite a bit, and 4 = extremely) and is divided into factors of 6 sub-areas: tension-anxiety, depression-dejection, anger-hostility, vigor-activity, fatigue-inertia, and confusion-bewilderment.

Sociability was evaluated using the sociability measuring model for juveniles developed by Lim and Lee that consists of 24 items, such as leadership, group life, being left out, sociability, expressiveness, and patience, each of which consists of four items [20]. In this sociability measurement model, the higher the sub-area score, the better the sociability, and the lower the sub-area score, the worse the sociability, except for the ‘being left out’ item.

Cognitive function was measured using the Korean version of the children’s version of the Stroop color and word test, which was originally developed by Golden et al. and adapted for Koreans by Shin and Park [21,22]. The Stroop color and word test consists of three conditions: word reading (Word), color reading (Color), and color-word reading (Color-Word). These conditions are used to evaluate the executive function of the frontal lobe that is involved in learning, attention, and problem-solving skills. The higher the recorded score, the better the cognitive function. Each condition consists of 100 items per page with five columns and 20 rows. In the word reading condition, “Red, Blue, Green” were randomly written in black ink. In the color reading condition, “XXXX” was written in blue, red, and green color ink, which was randomly assigned. The color-word condition consisted of 100 colored words that were written using the same word and color or a different word and color. The subject was asked to read the words aloud within 45 s and to read as many as possible under each condition (word, color, and color-word). In this study, the raw score of the subject that was correctly read aloud for each condition within the allocated time limit was recorded.

### 2.3. Taekwondo-Based Training Intervention Method

TKD-based training consisted of a training session of 60 min: 5 min of warmup and cool-down and 50 min of the main exercise. This session was performed once a week at 50–80% HRmax for 16 weeks, as shown in Table 2. All interventions were conducted by a TKD expert instructor by demonstration and coaching, and the main exercise consisted of 10 min of basic fitness such as shuttle runs, the Burpee test, and leapfrog, 5 min of six basic TKD motions and body punching in the horse-riding stance, 10 min of Poomsae from the 1st to 8th chapters, 10 min of kicking sessions with basic kicking and steps as well as mitt kicks, and 15 min of Taekwon gymnastics.

### 2.4. Statistical Analyses

The mean and standard deviation of all variables were calculated by SPSS Version 23.0 for Windows (IBM Corp., Armonk, NY, USA). Two-way repeated measures analysis of variance (ANOVA) was subsequently performed to investigate the differences in the dependent variable group (between the control and TKD groups) and time (before and after intervention). Independent and dependent *t*-tests were also conducted to identify statistically significant interactions. Statistical significance was set at 0.05.

## 3. Results

### 3.1. Changes in Physique and Fitness

Changes in physique (height, weight, BMI, and percentage of body fat) and fitness (VO_2_max, grip strength, back strength, sit-and-reach, Sargent jump, and Stork test) before and after the intervention in the control and TKD groups are shown in Table 3. Two-way repeated measures ANOVA for physique and fitness revealed a significant difference in the interaction for the Stork test score (*F* = 5.027, *p* = 0.033) between the intervention time (before and after) and groups (control and TKD). According to the post-hoc test, the Stork test score was not significantly different before and after the intervention in the control group. In contrast, the TKD group showed significantly improved sit-and-reach scores before and after the intervention (*p* < 0.05); however, except for the Stork test, there were no significant differences in terms of height, weight, BMI, percentage of body fat, VO_2_max, grip strength, back strength, sit-and-reach, or Sargent jump (*p* > 0.05).

### 3.2. Changes in Mood State

Changes in the mood state before and after the intervention in the control and TKD groups are shown in Table 4. Two-way repeated measures ANOVA for mood state revealed no significant interaction between intervention time (before and after) and group (control and TKD) for tension (*F* = 7.127, *p* = 0.012), depression (*F* = 6.964, *p* = 0.013), and vigor (*F* = 7.258, *p* = 0.012). Post-hoc tests showed no significant difference before and after the intervention in the control group. In contrast, the TKD group showed a significant reduction in tension and depression after the intervention and a significant increase in the vigor score (*p* < 0.05). Furthermore, after the intervention, the TKD group had a significantly higher vigor score than did the control group (*p* < 0.05). However, there were no significant differences in the scores for anger, fatigue, or confusion (*p* > 0.05).

### 3.3. Changes in Sociability

Changes in sociability before and after the intervention in the control and TKD groups are shown in Table 5. Two-way repeated measures ANOVA for sociability revealed a significant interaction for being left out (*F* = 10.036, *p* = 0.004) and sociability (*F* = 5.090, *p* = 0.032) between the intervention time (before and after) and group (control and TKD group). Post-hoc tests showed no significant difference before and after the intervention in the control group. In contrast, the TKD group showed a significantly lower ‘being left out’ score after than before the intervention and a significantly higher sociability score (*p* < 0.05). However, there was no significant difference in terms of leadership, group life, patience, or expressiveness (*p* > 0.05).

### 3.4. Changes in Cognitive Function

Changes in cognitive function before and after the intervention in the control and TKD groups are shown in Table 6. Two-way repeated measures ANOVA for cognitive function showed no significant interaction for the word (*F* = 0.971, *p* = 0.333), color (*F* = 2.301, *p* = 0.140), or color-word (*F* = 0.260, *p* = 0.614) tests between the intervention time (before and after) and group (control and TKD).

## 4. Discussion

Numerous recent studies have proved that regular physical activity such as exercising is the most effective, safe, accessible, and inexpensive strategy for skeletal development [23] and is essential for the improvement of body composition and health-related fitness in children and adolescents [24,25]. TKD training is also expected to have a positive effect on growth and development, as well as improve the general fitness, such as body composition, aerobic capacity, strength, power, and flexibility in both children and adolescents [11,13,14].

However, the present study revealed no significant difference in any of the variables, except for the Stork test score, related to physique and physical fitness, indicating that these results are from the weekly exercise frequency of TKD training. According to the American College of Sports Medicine guidelines, exercise is required 3–5 times a week or at least more than three times a week (at least more than twice a week for flexibility) in order to improve various aspects of fitness, such as cardiorespiratory endurance, strength and muscular endurance, and flexibility [26]. Furthermore, by reviewing 573 exercise-related papers for evidence of improvements in bone mineral accumulation, which is closely associated with growth in children and adolescents, Hind and Burrows reported that a frequency of exercise of three sessions per week was effective [27].

Recent studies have reported improvement in fitness and body composition in response to TKD training and suggested an exercise frequency of at least twice a week [14,28]. Kim et al. reported that TKD training twice a week for 12 weeks in female adolescents led to improvements in isokinetic strength, standing long jump, and sit-and-reach performance, as well as a significant reduction in the percentage of body fat and fat body mass [14].

Furthermore, our study showed that five sessions of TKD training per week were effective in improving the cardiorespiratory endurance (VO_2_max) in children [28]. TKD training intervention once a week does not appear to induce sufficient stimulation to develop the physique and improve fitness. The Stork test, which determines balance, demonstrated significant improvements after 16 weeks of TKD training. These results support those of previous studies that reported an improvement in balance following TKD training, indicating that the unique motions associated with TKD effectively improve balance [29,30].

Pons et al. reported that TKD includes various movements, such as jumping, gymnastics, and weight shifting and kicking exercises, and indicated that these highly dynamic movements can work effectively to improve the balance [30]. Furthermore, previous studies have reported that frequent jumps and spinning kicks in TKD training can stimulate the development of the vestibular system, thus supporting the present findings.

Multiple previous studies have reported that regular exercise is associated with physiological benefits, including an improvement in fitness and growth and development of the body as well as mental and social development in children during their major growth periods [31,32]. For example, Biddle and Asare suggested a relationship between physical activity and mental health in young people because physical activity can alleviate anxiety, although specific evidence for this relationship is limited [31]. Garcia et al. further reported that there is a close relationship between moderate-to-vigorous physical activity and social factors such as sociability in children [32].

Notably, it has been reported that participation in TKD training during childhood can be effective for emotional and social development by alleviating anxiety, promoting independence and leadership, and controlling aggression [15,33]. In the present study, we examined the changes in mood state and sociability in response to TKD training in children from multicultural families in Korea using a sociability questionnaire that examined the POMS, leadership, group life, being left out, patience, sociability, and expressiveness.

The present study showed that TKD led to significant reduction in tension and depression scores (sub-variables of POMS) and significant increases in the vigor score. Additionally, the ‘being left out’ score, a sub-variable of sociability, was significantly reduced, while the sociability score was significantly increased. This suggests that TKD training can be effective in improving the mood state and developing sociability in children from multicultural families and supports earlier studies that suggested that TKD exercise improved mood state, and that traditional TKD training was effective in enhancing sociability of adolescents [34,35,36]. For example, Toskovic reported that dynamic TKD practice induced a positive change in the mood state of 20 university students who showed significant improvements in tension, depression, anger, fatigue, confusion, and vigor, which are all sub-variables of POMS [35].

Trulson assigned juvenile delinquents to a traditional TKD group and a modern martial art (martial art as a competitive sport) group and trained them for six months [36]. The results revealed that the TKD group showed reduced aggression and anxiety in addition to improvements in social ability and self-esteem, indicating that TKD is based on self-control and self-defense while featuring key characteristics such as respect, humility, responsibility, perseverance, and honor [34,36]. This study also suggested that the moral cultural effect of TKD training could work effectively to improve the mood state and sociability in children from multicultural families.

In contrast, despite some previous studies that reported improvements in cognitive function secondary to regular TKD training [37,38], we observed no significant changes in the Stroop color and word test score, which were the assessment scales for cognitive function used in this study. It is believed that the lack of weekly TKD training would not lead to an improvement in aerobic fitness and fail to induce increases in neuroplasticity-related growth factors such as insulin-like growth factor (IGF)-1, vascular endothelial growth factor (VEGF), and brain-derived neurotrophic factor (BDNF). The fact that these factors were not directly examined represents a key limitation of the present study. Previous studies have reported that increased expression of neurotrophic and growth factors during exercise training is likely to contribute to improvements in cognitive function, although the specific mechanisms involved are not clearly understood [39,40], thus verifying the fact that the improvement in aerobic fitness is related to the increased expression of neuroplasticity-related growth factors [41,42,43]. In particular, IGF-1, which regulates the expression of VEGF and BDNF, showed a significant positive correlation with the level of aerobic fitness [43] and our own previous study showed that five sessions of TKD training for 16 weeks was effective in improving the cognitive function as it resulted in improved aerobic fitness (VO_2_max) and increased levels of serum IGF-1, VEGF, and BDNF [28].

This study has additional limitations. First, the number of subjects was small as it was a pilot study in a single institution. Korean discrimination against foreigners is based on the economic power of the corresponding country as lower economic power is linked to higher neglect, discrimination, and exclusivism [44], and the subjects in this study were children from Asian multicultural families despite the fact that there is racial inequality [45] such that children from Arab and South-East Asian multicultural families also have learning disabilities due to the lack of positive educational feedback compared with children from multicultural white (US and Great Britain) families. Second, sociodemographic characteristics of the parents of the subjects such as occupation and economic statuses could not be considered even though it is reported that psychosocial adaptation such as social withdrawal, depression/anxiety, and behavior problems such as delinquency and aggression are significantly affected by the degree of social relation network and economic status (monthly income) of parents in multicultural Korean children [46]. Third, it is hard to suggest the exact mechanism of how TKD training, despite the weekly frequency, was effective in improving the mood state and sociability, and it is hard to assert whether these positive effects are from regular participation in a physical activity or TKD training alone.

## 5. Conclusions

Our data indicate that participation in regular TKD training can be effective in improving the mood state and developing sociability in children from multicultural families. Within this cohort, however, TKD training did not result in significant changes in the physique, cardiorespiratory endurance, strength, power, flexibility, and cognitive function. Future studies are needed to investigate the positive effects of TKD training based on socio-economic status and cultural data (skin color, occupation, economic level such as monthly income, and national power and economic power of the country of origin) as variables, while simultaneous using latest technique such as functional magnetic resonance imaging for neurotransmitters and stress hormones that are involved in control of the mood, and by using a normal exercise group such as aerobic exercises as the control group.

## Figures and Tables

**Table 1:** Physical characteristics of the subjects at baseline.

Group Variables	Control (*n* = 15)	Taekwondo (*n* = 15)	*p* Value *
Gender (boys/girls)	9/6	9/6	
Age (years)	11.40 ± 0.63	11.53 ± 0.64	0.571
School grades (unit)	5.40 ± 0.63	5.47 ± 0.64	0.776
Height (cm)	148.79 ± 9.79	149.86 ± 8.14	0.746
Weight (kg)	46.08 ± 11.57	45.81 ± 12.48	0.952
Body mass index (kg/m^2^)	20.47 ± 3.34	20.15 ± 4.26	0.821
Body fat (%)	18.80 ± 6.35	16.79 ± 7.61	0.438

Data are presented as mean ± standard deviation. * *p* value as determined using the independent *t*-test for each of the two groups at baseline.

**Table 2:** Taekwondo-based training programs.

Classification		Main Activity	Time
Warm-up		Stretching	5 min
Main exercise	Basic fitness	Shuttle run, Burpee test, Sargent jump, leapfrog	10 min
	Taekwondo basic motion	Close stance, Parallel stance, Riding stance, Forward stance, Forward inflection stance, Backward inflection stance, body punch in horse-riding stance	5 min
	Poomsae	Taegeuk 1–8 chapter	10 min
	Kicking	Basic kicking (front kick, turning kick, side kick, downward kick), mitt kick and so on	10 min
	Taekwon gymnastics *	2 music-based gymnastics	15 min
Cool-down		Stretching	5 min

* Taekwon gymnastics: gymnastic motions in Taekwondo that are adjusted to a musical rhythm.

**Table 3:** Changes in physique and physical fitness.

Group Variables	Control (*n* = 15)		Taekwondo (*n* = 15)		Time × Group Interaction	
	Before	After	Before	After	*F*	*p*
Height (cm)	148.79 ± 9.79	149.11 ± 9.66	149.86 ± 8.14	150.51 ± 8.24	2.483	0.126
CV	0.07	0.06	0.05	0.05		
Weight (kg)	46.08 ± 11.57	46.33 ± 11.02	45.81 ± 12.48	45.95 ± 11.45	0.088	0.768
CV	0.25	0.24	0.25	0.21		
BMI (kg/m^2^)	20.47 ± 3.34	20.57 ± 3.17	20.15 ± 4.26	20.05 ± 3.72	0.814	0.375
CV	0.16	0.15	0.21	0.19		
Body fat (%)	18.80 ± 6.35	18.73 ± 5.99	16.79 ± 7.61	16.73 ± 7.15	0.003	0.960
CV	0.34	0.32	0.45	0.43		
VO_2_max (ml/kg/min)	28.77 ± 5.86	28.86 ± 5.90	28.45 ± 5.50	28.78 ± 5.38	0.371	0.547
CV	0.20	0.20	0.19	0.19		
Grip Strength (kg)	17.17 ± 4.26	17.50 ± 4.32	17.29 ± 4.67	18.07 ± 4.63	1.130	0.297
CV	0.25	0.25	0.27	0.26		
Back Strength (kg)	38.67 ± 8.40	39.59 ± 8.98	39.83 ± 9.43	40.85 ± 10.16	0.024	0.877
CV	0.22	0.23	0.24	0.25		
Sit-and-reach (cm)	8.29 ± 7.03	8.38 ± 6.92	8.18 ± 6.24	8.20 ± 5.71	0.070	0.794
CV	0.85	0.83	0.76	0.70		
Sargent jump (cm)	30.47 ± 7.41	30.60 ± 7.64	33.80 ± 7.89	34.47 ± 7.92	0.558	0.461
CV	0.24	0.25	0.23	0.23		
Stork test (point)	43.53 ± 29.61	44.60 ± 29.13	46.33 ± 33.94	51.93 ± 35.19 ^#^	5.027	0.033 *
CV	0.68	0.65	0.73	0.68		

Data are presented as mean ± standard deviation. CV, coefficient of variation; ^#^ significant difference before and after intervention within the group (*p* < 0.05); * *p* < 0.05.

**Table 4:** Changes in mood state.

Group Variables	Control (*n* = 15)		Taekwondo (*n* = 15)		Time × Group Interaction	
	Before	After	Before	After	*F*	*p*
Tension (score)	1.92 ± 0.57	1.95 ± 0.68	1.89 ± 0.67	1.57 ± 0.54 ^#^	7.127	0.012 *
CV	0.30	0.35	0.35	0.34		
Depression (score)	2.20 ± 0.68	2.31 ± 0.66	2.20 ± 0.73	1.93 ± 0.70 ^#^	6.964	0.013 *
CV	0.31	0.29	0.33	0.36		
Anger (score)	1.61 ± 0.52	1.63 ± 0.57	1.61 ± 0.47	1.61 ± 0.46	0.012	0.913
CV	0.32	0.35	0.29	0.28		
Vigor (score)	1.61 ± 0.67	1.72 ± 0.59	1.61 ± 0.54	2.20 ± 0.53 ^#,+^	7.258	0.012 *
CV	0.42	0.34	0.34	0.24		
Fatigue (score)	1.63 ± 0.63	1.64 ± 0.56	1.59 ± 0.56	1.57 ± 0.51	0.979	0.331
CV	0.39	0.34	0.35	0.32		
Confusion (score)	1.88 ± 0.55	1.91 ± 0.49	1.89 ± 0.51	1.89 ± 0.46	0.028	0.867
CV	0.29	0.26	0.27	0.24		

Data are presented as mean ± standard deviation. CV, coefficient of variation; ^#^ significant difference before and after intervention within the group (*p* < 0.05); ^+^ significant difference between the control and Taekwondo group within the time period (*p* < 0.05); * *p* < 0.05.

**Table 5:** Changes in sociability.

Group Variables	Control (*n* = 15)		Taekwondo (*n* = 15)		Time × Group Interaction	
	Before	After	Before	After	*F*	*p*
Leadership (score)	2.38 ± 0.45	2.41 ± 0.66	2.41 ± 0.61	2.45 ± 0.54	0.004	0.953
CV	0.19	0.28	0.25	0.22		
Group life (score)	3.05 ± 0.64	3.05 ± 0.57	3.07 ± 0.57	3.09 ± 0.51	0.089	0.768
CV	0.21	0.19	0.19	0.16		
Being left out (score)	2.17 ± 0.57	2.23 ± 0.61	2.21 ± 0.79	1.84 ± 0.65 ^#^	10.036	0.004 **
CV	0.26	0.27	0.36	0.36		
Patience (score)	2.50 ± 0.78	2.51 ± 0.80	2.49 ± 0.70	2.59 ± 0.71	0.473	0.497
CV	0.31	0.32	0.28	0.27		
Sociability (score)	2.79 ± 0.79	2.75 ± 0.80	2.70 ± 0.69	2.99 ± 0.64 ^#^	5.090	0.032 *
CV	0.28	0.29	0.26	0.21		
Expressiveness (score)	2.78 ± 0.71	2.71 ± 0.64	2.73 ± 0.58	2.89 ± 0.66	1.918	0.177
CV	0.26	0.24	0.21	0.23		

Data are presented as mean ± standard deviation. CV, coefficient of variation; ^#^ significant difference before and after intervention * *p* < 0.05; ** *p* < 0.01.

**Table 6:** Changes in cognitive function.

Group Variables	Control (*n* = 15)		Taekwondo (*n* = 15)		Time × Group Interaction	
	Before	After	Before	After	*F*	*p*
Word test (score)	71.68 ± 16.52	72.75 ± 16.10	73.28 ± 14.74	76.16 ± 13.25	1.227	0.277
CV	0.23	0.22	0.20	0.17		
Color test (score)	54.86 ± 11.11	55.38 ± 8.87	55.12 ± 9.93	57.63 ± 9.19	1.663	0.208
CV	0.20	0.16	0.18	0.16		
Color-Word test (score)	36.40 ± 8.24	38.27 ± 7.77	36.80 ± 7.02	39.33 ± 7.39	0.260	0.614
CV	0.23	0.20	0.19	0.19		

Data are presented as mean ± standard deviation. CV, coefficient of variation.
